# Relationship of Hair Cortisol Concentration With Perceived and Somatic Stress Indices: Cross-Sectional Pilot Study

**DOI:** 10.2196/63811

**Published:** 2025-06-11

**Authors:** Sharon H Bergquist, Danyang Wang, Brad Pearce, Alicia K Smith, Allison Hankus, David L Roberts, Miranda A Moore

**Affiliations:** 1 Department of Medicine, School of Medicine, Emory University, 1365 Clifton Rd, Atlanta, GA, 30322, United States, 1 4047786417; 2 Rollins School of Public Health, Emory University, Atlanta, GA, United States; 3 Department of Gynecology and Obstetrics, School of Medicine, Emory University, Atlanta, GA, United States; 4 Department of Family and Preventive Medicine, School of Medicine, Emory University, Atlanta, GA, United States

**Keywords:** hair cortisol concentration, perceived stress, somatic stress, resilience, physiological stress

## Abstract

**Background:**

Hair cortisol is an emerging biomarker of chronic stress. However, the psychological and physiological aspects of chronic stress that are reflected in hair cortisol concentration (HCC) have not been fully determined. Since physiological responses to stress do not always align with how stress is perceived, we conducted this study to evaluate whether HCC correlates with neuroendocrine stress indicators or stress perceptions.

**Objective:**

This study aimed to evaluate whether subjective (Perceived Stress Scale and Connor-Davidson Resilience Scale) and objective (plasma cortisol/dehydroepiandrosterone-sulfate [DHEA-S] and cortisol/high-sensitivity C-reactive protein) determinants of stress and resilience correlate with HCC.

**Methods:**

In this cross-sectional pilot validity study, scatter plots and Spearman correlation coefficients were used to measure the direction and magnitude of the relationship between stress and resilience measures among 51 predominantly male participants. In a subset (n=24), we performed a step-wise regression modeling approach to isolate the association of perceived and somatic stress on hair cortisol.

**Results:**

Bivariate correlations showed a weak inverse association of HCC with Perceived Stress Scale (Spearman correlation ρ=−0.14, *P*=.52) and a stronger positive association with somatic neuroendocrine stress indices cortisol/DHEA-S (ρ=0.24, *P*=.25) and cortisol/high-sensitivity C-reactive protein (ρ=0.21, *P*=.35). In linear regression models, HCC showed the strongest association with cortisol/DHEA-S (*r*^2^=0.10, *P*=.13, 1.01^β^ 1.01, 95% CI 0.99‐1.01). This relationship remained when age, gender, hair washing frequency, hair dye or bleach use, diabetes mellitus, obesity, cardiovascular disease, anxiety, medication use, and endocrine disorders were considered.

**Conclusions:**

Our results do not indicate a statistically significant association (at the *P*<.05 threshold) between HCC and stress perception or somatic measures of neuroendocrine response.

## Introduction

Stress and its related comorbid diseases are responsible for a large proportion of health care expenditure [1,2] and disability worldwide [3]. Psychiatric disorders, including stress-related disorders, are predicted to be second only to ischemic heart disease as the leading cause of disability by 2020 [4]. Stress can be defined as the self-perception that an individual does not have the resources to cope or respond to a threat [5]. The duration of stress exposure is a key determinant of the disease risk attributed to stress. Acute stress is an adaptive response to a challenge and is generally not harmful to health [6]. However, when stress is severe, or experienced continuously or repeatedly, it can lead to psychological and physical illness [5,7]. While the detrimental effects of chronic stress on health are well-recognized, measuring stress burden remains challenging [8]. Biomarkers of chronic stress reactivity are needed for early risk determination and targeted risk management in clinical practice [9].

Current instruments used for assessing stress burden include subjective and objective measures, each of which has limitations. Subjective self-assessments rely on a conscious perception of stress, which can be influenced by genetic, developmental, cultural, social, and resilience factors. Hence the perception of the quality and intensity of a stressor is not consistently correlated with physiologic stress reactivity [7,8]. The commonly used objective assessment of stress is via measurement of the glucocorticoid cortisol, the downstream effector released by the neuroendocrine hypothalamus-pituitary-adrenal (HPA) axis. Unfortunately, capturing long-term cortisol secretion is methodologically challenging. Traditional measures of salivary and plasma cortisol provide insight into cortisol level at a single point in time [9], and are subject to diurnal fluctuation [10]. Cortisol sampling over 1 day can be accomplished with a 24-hour urine collection. In addition to being logistically burdensome, this short time interval also fails to capture long-term cortisol output.

Since cortisol is incorporated into growing hair, hair is emerging as a novel matrix for measuring retrospective cortisol secretion over months [9,11,12]. Each 1 cm hair segment, beginning from the proximal end, approximates a month’s cortisol production. While accumulating evidence supports the validity of hair cortisol concentration (HCC) as an index of long-term systemic cortisol secretion and its reliability across repeated assessments [10,11], 2 central gaps remain in its clinical applicability [9-11]. The first is determining the covariates that can influence HCC. Research has identified age, sex, hair washing frequency, hair treatment, and oral contraceptive use as relevant covariates [12]. Health conditions and other medications can also influence HCC [10]. The second is understanding the stress-related determinants of HCC [12]. Thus far, the correlation between validated measures of perceived stress and HCC has been inconsistent [9,12,13]. Psychological resilience has been shown to moderate the indirect association of perceived stress with illness severity via HCC [14] but little research is available on the relationship between HCC and physiologic stress indices.

We hypothesize that the strength of the correlation of HCC with somatic measures of stress will be greater than with psychologic measures of stress. We additionally speculate that the relationship of HCC with somatic stress constructs will be stronger among constructs that take into account neuroendocrine resilience factors and neuroinflammatory responses. Current evidence indicates that during the stress response, dehydroepiandrosterone (DHEA) and its sulfate ester, dehydroepiandrosterone-sulfate (DHEA-S), collectively referred to as DHEA(S), are cosynthesized with cortisol and released from the adrenal glands [15]. Several lines of evidence suggest DHEA has anabolic and neuroprotective effects that antagonize cortisol [16,17], and the ratio of cortisol to DHEA(S) has been considered an index of “net steroid activity” [18]. Cortisol/DHEA(S) may be a better predictor of stress outcomes than either hormone alone [18,19] and a higher ratio of cortisol/DHEA(S) has been shown to predict a higher risk of disease [18,20].

Another factor potentially moderating the association between stress reactivity and HCC is the chronicity of stress, which has been proposed to result in glucocorticoid receptor resistance [21] and consequent failure to down-regulate the inflammatory response [21]. The ratio of cortisol to high-sensitivity C-reactive protein (hs-CRP) has been examined as an alternate index that captures the integrity of homeostatic regulation between the HPA axis and inflammatory processes [22].

Investigating levels of HCC, cortisol, DHEA-S, and hs-CRP simultaneously may provide important clinical insights; although this has rarely been done in clinical studies [23]. The first aim of this study is to examine the interrelation between HCC and subjective measures of stress (Perceived Stress Scale [PSS]) and resilience (Connor Davidson Resilience Scale [CD-RISC]), and different objective measures of neuroendocrine stress reactivity and inflammatory balance (cortisol/DHEA-S, cortisol/hs-CRP). The secondary aim is to assess the health-related and other covariates that can influence HCC.

## Methods

### Recruitment

We conducted a prospective, cross-sectional cohort, pilot study of 51 participants from patients seen at the executive health program at Emory University in Atlanta, Georgia between May 23, 2017 and October 19, 2017. The executive health program provides comprehensive health examinations primarily to senior-level corporate executives. During the nursing intake process, the 499 patients seen in the clinic in the time period were invited to participate, and written informed consent was obtained and the nature of the procedures was fully explained. Participants completed 2 survey assessments (totaling approximately 10 min) and provided a 2 mL tube of blood in addition to providing clinical histories and undergoing tests obtained during routine care. Furthermore, 24 participants also agreed to provide a pencil-wide hair sample used for hair cortisol analysis. The patients did not receive their study results and there were no follow-ups to the initial study data collection.

### Ethical Considerations

Participation was voluntary and patients were notified that participation would not impact their clinical care. No payment was offered for participation. The protocol for this study was approved by the Emory University Institutional Review Board (IRB 00092476). This study was conducted in accordance with the latest version of the Declaration of Helsinki. All data were deidentified for publication. During the nursing intake process, patients were invited to participate, and written informed consent (Multimedia Appendix 1) was obtained, and the nature of the procedures was fully explained.

### Psychological Stress Measures

The PSS 10-item designed by Cohen et al [24] was used for assessing perceived stress over the past month. This psychometrically validated 10-item self-reported questionnaire is the most widely used psychological instrument for measuring the degree to which an individual appraises situations in one’s life as stressful. Each item is forward or reverse scored from 0 to 4 based on the experienced frequency of each item. Scores on the PSS can range from 0 to 40 with higher scores indicating higher perceived stress. The US population probability sample validated mean PSS value was 12.1 (SD 5.1) for males overall and 12.6 (SD 6.1) for males aged 45‐54 years [25,26]. The CD-RISC 25-item has robust psychometric properties for assessing resilience, viewed as a measure of stress-coping ability [27]. It is comprised of 25 items, each rated on a 5-point scale (0‐4), with higher scores reflecting greater resilience. The total possible scores range from 0 to 100. Scores were categorized by quintile based on results of the general US population, with the lowest quintile of resilience Q1 (0‐73), Q2 (74-82), Q3 (83‐90), and Q4 (91-100) [27]. Furthermore, 2 additional questions, rated on a 1‐10 Likert scale, were also obtained: (1) “How would you rate your stress level?” and (2) “How would you rate how you respond to stress?”

### Cortisol, DHEA(S), and Hs-CRP

Blood samples were obtained early morning (6:35 AM to 9:36 AM) in all participants and timing of blood drawn was recorded to reduce cortisol variability from diurnal cortisol rhythms. Blood was also collected in the fasting state to avoid the potential of meal effect on cortisol. For plasma cortisol and DHEA(S), a 2 mL serum separator tube was processed. Plasma cortisol and DHEA are reported in nanograms per milliliter (ng/mL). DHEA(S) is reported in micrograms per milliliter (μg/mL). Plasma cortisol was determined by enzyme-linked immunosorbent assay (ELISA) per the manufacturer protocol (IBL America IB79343). The cortisol assay dynamic range was 10‐800 ng/mL with a sensitivity of 3.79 ng/mL. Plasma DHEA and DHEA(S) were determined by discrete ELISAs per the manufacturer protocols (IBL IB79332; IBL IB79342). The DHEA assay dynamic range was 0.3‐30 ng/mL with a sensitivity of 0.07 ng/mL. The DHEA(S) assay dynamic range was 0.03‐10 µg/mL with a sensitivity of 0.002 µg/mL. Hs-CRP was processed as part of the routine physical examination laboratory assessment by Emory Medical Laboratory, the fully accredited and licensed clinical laboratory operating within Emory Healthcare.

### Hair Collection and Analysis

Hair samples were collected from a subset of participants (n=24), limited by willingness to participate and ability to contribute sufficient hair volume in this predominantly male cohort. Those who provided hair samples were given a questionnaire that solicited the frequency of hair washing; use of scalp treatments; and use of hair dyes, bleaching products, permanent straighteners, or curling products in the previous 3 months. A pencil-wide distribution (approximately 50 hairs) was cut with scissors as close as possible to the posterior vertex scalp to yield the lowest intraindividual coefficient of variation for cortisol concentration [28]. The hair sample was placed in a collection kit, with the root end indicated. Hair length was measured from the root end and isolated. The samples were weighed, washed in isopropanol, dried, and transferred to metal bead lysing matrix tubes (MP 6925‐100). The transferred sample weight was recorded. The lysing tubes were run for cycles of 40s in a grinding machine (BIO101 Savant FastPrep FP120 [Savant Instruments]) until each sample reached uniform powder consistency. Upon grinding completion, cortisol was extracted in methanol overnight. The samples were centrifuged to pellet the powdered hair and the supernatant was isolated. The extracted cortisol supernatant was placed in a vacuum evaporator (Savant DNA Speed Vac DNA120 [Savant Instruments]) set at RT dry for 100 min to completely remove methanol from the sample. The cortisol extract was reconstituted in ELISA assay diluent (Salimetrics PN8005). Cortisol was measured via immunoassay (Salimetrics 1‐3002), in a single batch, using internal positive controls as standards, to circumvent potential interassay variability among immunoassays. The Salimetrics cortisol assay dynamic range was 0.3‐3 µg/dL with a sensitivity of 0.007 µg/dL.

Demographic and clinical characteristics, including the presence of mental and physical conditions, were obtained from the medical record.

### Statistical Analysis

Descriptive statistics analyses were performed to obtain mean (SD) or number (percentage) for sociodemographic characteristics, stress variables, and medical conditions and use of medications that may alter cortisol production. As many of our variables are clinically limited to a specific range, the following variables contained outliers and were truncated: plasma DHEA (2 observations truncated at 15.0 ng/mL) and plasma DHEA(S) (2 truncated at 5 μg/mL). In addition, normality tests were performed to determine if each variable was well-modeled by normal distributions. Based on this analysis, plasma DHEA and plasma DHEA(S) were log-transformed for analysis. We used scatter plots and Spearman correlation coefficients (ρ) to measure the direction and magnitude of the relationship between stress and resilience measures.

To isolate the impact of perceived and somatic stress on HCC from the impact of other factors impacting HCC, we used a step-wise regression modeling approach. As a first step, we used simple linear regression modeling of each perceived and somatic neuroendocrine stress construct regressed on HCC to determine which variables had the strongest independent association. Next, we used multivariate regression modeling to calculate fully adjusted associates controlling for factors impacting HCC. Based on previous studies, the covariates included were age, gender, frequency of hair washing, use of hair dyes or bleach, topical and systemic medication (dichotomous indicator of any use), prediabetes or diabetes mellitus, obesity, subclinical or clinical cardiovascular disease, anxiety, and endocrine disorders [10,12]. Race was not included in our model as there was limited variability in the sample.

Other mental health conditions (depression, post-traumatic stress disorder, and bipolar illness) were also queried but not included in the regression model due to an insufficient number of observations with known data among participants who provided hair samples. Exponentiation, based on the variable form, of the β-coefficient with 95% CIs and *t* values were calculated. We performed sensitivity analyses for the variables in our models to determine the minimum effect size that could yield a statistically significant result with a significance level of 0.05 and power of 0.80 for our sample size using Cohen *f*^2^ measures. All study variables were available for all study participants; thus, we had no missing data to exclude. All data analyses were conducted using StataCorp LLC StataSE 16 software [29].

## Results

### Demographic and Stress Characteristics

The average age of the full sample of participants in our study was 53.7 (SD 8.8 y; range 37‐77 y, Table 1). The majority were white (48/51, 94%) males (46/51, 92%). Our sample PSS values were slightly lower than the US national average at 11.7 (SD 5.4; range 3.0‐28.0). The mean CD-RISC score in our sample was 82.6 (SD 12.4; range 46.0‐100.0), which was higher than the mean score of 80.4 (SD 12.8) in the US general population [28]. Mean levels of cortisol, DHEA, and DHEA(S) were 225.3 (SD 67.6) ng/mL, 4.9 (SD 3.2) ng/mL, and 2.2 (SD 1.2) μg/mL, respectively.

Demographic, stress, and clinical characteristics were not significantly different in the 24 participants in whom sufficient hair volume was available for analysis than in the full sample of participants (Table 1). The average HCC was 32.32 (SD 45.50; range 1.29‐202.41) ng/g hair. Among the 24 participants, the average age was 54.8 (SD 10.1; range 39‐77) years. Furthermore, 83% (20/24) were males and 95% (23/24) were White. The average PSS value was 11.9 (SD 6.0; range 3.0‐28.0). The mean CD-RISC score was 85.0 (SD 14.0; range 46.0‐100.0). Mean levels of cortisol, DHEA, and DHEA(S) were 227.2 (SD 64.8) ng/mL, 4.6 (SD 3.1) ng/mL, and 2.2 (SD 1.0) μg/mL, respectively (Table 1).

**Table 1.** Demographic, stress, and clinical characteristics (this prospective, cross-sectional cohort, pilot study includes 51 patients seen in the executive health clinical program at Emory Healthcare in Atlanta, Georgia between May 23, 2017 and October 19, 2017).

Characteristics	Full sample (n=51)		Regression sample (n=24)		Nonregression sample (n=27)		*P* value^h^
	Statistical value	Range	Statistical value	Range	Statistical value	Range	
Age (years), mean (SD)	53.7 (8.8)	37-77	54.8 (10.1)	39-77	52.8 (7.6)	37-66	.43
Sex, n (%)							
Female	5 (10)	—^i^	4 (17)	—	1 (4)	—	.18
Male	46 (90)	—	20 (83)	—	26 (96)	—	—
Race, n (%)							
White	48 (94)	—	23 (96)	—	25 (93)	—	≥.99
Other	3 (6)	—	1 (4)	—	2 (7)	—	—
Self-rated stress (Likert scale; n=50), mean (SD)	6.2 (2.1)	2-10	6.3 (2.2)	2-10	6.1 (1.9)	3-10	.71
Self-rated response to stress (Likert scale; n=50), mean (SD)	2.7 (1.5)	1-8	2.6 (1.7)	1-8	2.9 (1.3)	1-6	.47
Perceived Stress Scale-10 (total score), mean (SD)	11.7 (5.4)	3-28	11.9 (6.0)	3-28	11.6 (5.0)	2-23	.86
Connor-Davidson Resilience Scale-25 (total score), mean (SD)	82.6 (12.4)	46-100	85.0 (14.0)	46-100	80.4 (10.5)	55-98	.19
Q1^j^ (0‐73), n (%)	11 (22)	—	4 (17)	—	7 (26)	—	.02
Q2^k^ (74-82), n (%)	12 (24)	—	3 (13)	—	9 (33)	—	—
Q3^l^ (83-90), n (%)	13 (26)	—	5 (21)	—	8 (30)	—	—
Q4^m^ (91-100), n (%)	15 (29)	—	12 (50)	—	3 (11)	—	—
Hair cortisol (ng/g; n=24), mean (SD)	32.3 (45.5)	1.3-203.4	32.3 (45.5)	1.3-202.4	—	—	—
Hair bleached or dyed, n (%)	6 (25; n=24)	—	5 (22; n=23)	—	—	—	—
Hair washing frequency (times/week), mean (SD)	6.3 (1.7; n=24)	1-7	6.2 (1.7; n=23)	1-7	—	—	—
Plasma cortisol (ng/ml), mean (SD)	225.3 (67.6)	102.0-366.6	227.2 (64.8)	114.1-330.2	223.7 (71.1)	102.0-366.6	.86
Plasma DHEA^n^ (ng/ml), mean (SD)	4.9 (3.2)	0.9-15.0^o^	4.6 (3.1)	0.9-15.0^o^	5.2 (3.3)	1.4-15.0^o^	.55
Plasma DHEA(S^p^) (μg/ml), mean (SD)	2.2 (1.2)	0.0-5.0^o^	2.2 (1.0)	0.1-3.8	2.2 (1.3)	0.0-5.0^o^	.80
Medical condition							
Obesity (BMI≥30 kg/m^2^), n (%)	13 (26)	—	7 (30)	—	6 (22)	—	.57
Prediabetes or diabetes mellitus, n (%)	12 (24)	—	10 (42)	—	2 (7)	—	.004
Subclinical or clinical cardiovasculardisease, n (%)	16 (31)	—	9 (38)	—	7 (26)	—	.37
Endocrine disorders, n (%)	5 (10)	—	2 (8)	—	3 (11)	—	≥.99
Generalized anxiety disorder, n (%)	5 (10)	—	3 (13)	—	2 (7)	—	.66
Sleep (h/night), mean (SD)	6.7 (1.0; n=19)	5-8	6.7 (1.0; n=8)	5-8	6.7 (1.0; n=11)	5-8	.93
Medications, n (%)							
Topical scalp medications							
Steroids	0 (0)	—	0 (0)	—	0 (0)	—	—
Nonsteroids	1 (2)	—	1 (4)	—	0 (0)	—	—
Oral medications							
OCP^q^ or HRT^r^	1 (2)	—	0 (0)	—	1 (4)	—	—
SSRI^s^ or SNRI^t^	1 (2)	—	1 (4)	—	0 (0)	—	—
Testosterone or other hormones	1 (2)	—	0 (0)	—	1 (4)	—	—

h *P* values for differences between the regression sample and nonregression sample, using *t* test, chi-square test, or Fisher exact test.

i Not applicable.

j Q1: quintile 1.

k Q2: quintile 2.

l Q3: quintile 3.

m Q4: quintile 4.

n DHEA: dehydroepiandrosterone.

o range after truncation.

p DHEA(S): collective term for dehydroepiandrosterone and dehydroepiandrosterone-sulfate.

q OCP: oral contraceptive pills.

r HRT: hormone replacement therapy.

s SSRI: selective serotonin reuptake inhibitor.

t SNRI: serotonin and norepinephrine reuptake inhibitor.

### Hair Cortisol and Its Relationship With Perceived and Physiologic Stress Reactivity Measures

**Figure 1.**
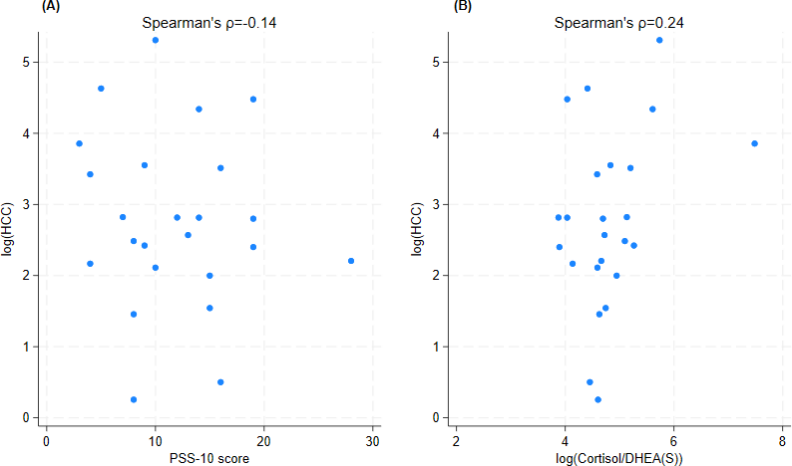
Correlation between log hair cortisol concentration and predictors. Log transformed hair cortisol concentration does not show a linear relationship with (A) Perceived Stress Scale-10 score but does show a weak to moderate linear correlation with (B) log transformed cortisol/DHEA(S) ratio. DHEA(S): collective term for dehydroepiandrosterone and dehydroepiandrosterone-sulfate; HCC: hair cortisol concentration; PSS: Perceived Stress Scale.

There was a significant inverse association between PSS and CD-RISC scores (ρ=−0.33, *P*<.05, Table 2). However, PSS and the ratio of PSS/CD-RISC showed weak inverse associations with HCC and did not reach statistical significance (ρ=−0.14 and −0.10, *P*=.52 and .63, respectively). The association was stronger between HCC and the neuroendocrine stress indices of cortisol/DHEA(S) (ρ=0.24, *P*=.25) and cortisol/hs-CRP (ρ=0.21, *P*=.35), but the strength of the positive association was weak to moderate and did not reach significance (Table 2 and Figure 1).

**Table 2.** Bivariate Spearman correlations between subjective and objective stress-related determinants (this prospective, cross-sectional cohort, pilot study includes 24 patients seen in the executive health clinical program at Emory Healthcare in Atlanta, Georgia between May 23, 2017 and October 19, 2017).

Stress-related determinants	1	2	3	4	5	6	7
PSS^a^-10 score	—^b^	—	—	—	—	—	—
CD-RISC-25^c^ score	−0.33^d^	—	—	—	—	—	—
PSS-10 or CD-RISC-25	0.96^e^	−0.54^e^	—	—	—	—	—
HCC^f^^g^	−0.14	−0.05	−0.10	—	—	—	—
Plasma cortisol	−0.08	0.07	−0.11	0.07	—	—	—
Cortisol/DHEA^h^^i^	−0.06	−0.05	−0.05	0.09	0.04	—	—
Cortisol/DHEA(S)^i^^j^	−0.04	−0.02	−0.03	0.24	0.33^d^	0.55^e^	—
Cortisol/hs-CRP^k^	−0.12	−0.09	−0.13	0.21	0.30^d^	−0.03	0.14

a PSS: Perceived Stress Scale.

b Not applicable.

c CD-RISC: Connor Davidson Resilience Scale.

d Significance level *P*<.05.

e Significance level *P*<.001.

f HCC: hair cortisol concentration.

g n=24.

h DHEA: dehydroepiandrosterone.

i Variable truncated.

j DHEA(S): collective term for dehydroepiandrosterone and dehydroepiandrosterone-sulfate.

k hs-CRP: high-sensitivity C-reactive protein.

This prospective, cross-sectional cohort, pilot study includes 24 patients seen in the executive health clinical program at Emory Healthcare in Atlanta, Georgia between May 23, 2017 and October 19, 2017.

In simple linear regression models using hair cortisol as the outcome variable and PSS, cortisol, cortisol/DHEA, cortisol/DHEA(S), and cortisol/hs-CRP as the respective predictor variables, HCC showed the strongest association with cortisol/DHEA(S) (*r*^2^=0.10, *F*_1,22_=2.51, *P*=.13, 1.01^β^ 1.01, 95% CI 0.998‐1.012, Table 3). A moderate positive relationship remained when age, gender, frequency of hair washing, use of hair dyes or bleach, prediabetes or diabetes mellitus, obesity, subclinical or clinical cardiovascular disease, anxiety, endocrine disorders, and medications were used as control variables (Table 3). In the fully adjusted model, each 1% increase in cortisol/DHEA(S) was associated with a 1% increase in HCC (1.01^β^ 1.00, 95% CI 0.996‐1.011, *t*_2_=1.00, *P*=.34). In addition, the control variables with the strongest association to hair cortisol were history of an endocrine disease, subclinical or clinical cardiovascular disease, male, shampoo frequency, and age.

In sensitivity analyses, the minimum effect size for yielding a statistically significant result with our sample size was Cohen *f*^2^ of 0.358 for simple linear regressions. Our observed Cohen *f*^2^ ranged from 0.001 to 0.114. For multiple linear regressions, the minimum Cohen *f*^2^ measuring the local effect size of cortisol/DHEA(S) that can be detected at 80% power is 0.412. The observed Cohen *f*^2^ for cortisol/DHEA(S) was 0.091, indicating a small effect.

**Table 3.** Simple and multiple linear regression analyses (this prospective, cross-sectional cohort, pilot study includes 24 patients seen in the executive health clinical program at Emory Healthcare in Atlanta, Georgia between May 23, 2017 and October 19, 2017).

Regression determinants	Log (HCC)^a^				
	β coefficient	e^β^ or 1.01^β^ (95% CI of e^β^ or 1.01^β^)	Regression *t* statistics^b^	*R* ^2^	Cohen *f*^2^
Simple linear regressions					
PSS-10^c^ score	−0.025	0.98 (0.89-1.07)	−0.58	0.02	0.02
CD-RISC-25^d^ score	−0.003	0.997 (0.96-1.034)	−0.16	0.001	0.001
Serum cortisol	0.001	1.00 (0.99-1.01)	0.30	0.004	0.004
log (Cortisol/DHEA)^e^^f^	0.26	1.00 (0.99-1.01)	0.48	0.01	0.01
log (Cortisol/DHEA(S^g^))	0.52	1.01 (0.998-1.01)	1.58	0.10	0.11
log (Cortisol/hs-CRP)^h^	0.33	1.00 (0.998-1.01)	1.42	0.09	0.10
Multiple linear regression					
log (Cortisol/DHEA(S))	0.33	1.00 (0.996-1.01)	1.00	—^i^	0.09
Hair washing frequency	0.05	1.05 (0.71-1.55)	0.28	—	0.01
Hair bleached or dyed	−0.28	0.80 (0.13-4.76)	−0.28	—	0.01
Age	0.01	1.01 (0.94-1.08)	0.33	—	0.01
Male	0.20	1.22 (0.08-18.09)	0.17	—	0.002
Prediabetes or diabetes	−0.89	0.41 (0.12-1.45)	−1.56	—	0.22
Obesity	−1.33	0.27 (0.07-1.05)	−2.12	—	0.41
Subclinical or clinical CVD^j^	0.18	1.197 (0.25-5.63)	0.26	—	0.01
Anxiety	−1.26	0.28 (0.02-3.83)	−1.06	—	0.10
Endocrine disorders	1.97	7.19 (0.51-101.38)	1.64	—	0.25
On medications	−1.52	0.22 (0.03-1.55)	−1.71	—	0.27

a HCC: hair cortisol concentration.

b The multiple linear regression has a single df for the entire model, that is, df=13.

c PSS-10: Perceived Stress Scale-10.

d CD-RISC-25: Connor Davidson Resilience Scale-25.

e DHEA: dehydroepiandrosterone.

f Variable truncated.

g DHEA(S): collective term for dehydroepiandrosterone and dehydroepiandrosterone-sulfate.

h hs-CRP: high-sensitivity C-reactive protein.

i Not applicable.

j CVD: cardiovascular disease.

## Discussion

### Principal Results

The primary aim of this study was to examine the relationship between HCC and subjective stress perception and neuroendocrine indicators derived from blood samples. We did not find a statistically significant association between HCC with somatic stress indices or with perceived stress at the *P*<.05 level. We did find several weak to moderate nonsignificant correlations, with a stronger relationship between HCC and other somatic indices of stress rather than with perceived stress. Interestingly, HCC showed the strongest positive association with cortisol/DHEA(S). While we hypothesized that somatic stress indicators would show a stronger association with HCC, our results did not support a significant difference. Of interest, we found weak evidence that neuroendocrine indices that include the modulating effect of resilience factors may complement HCC as a clinical tool for measuring chronic stress burden.

Although HCC is emerging as a useful biomarker of chronic stress, the elements of cortisol production and stress response regulation reflected in HCC remain undetermined. Previous investigations of stress-related determinants of HCC have focused primarily on subjective self-evaluations of stress rather than on somatic neuroendocrine parameters of stress reactivity. To the best of our knowledge, Qiao et al [23] have conducted the only study evaluating HCC and DHEA/cortisol simultaneously, with all measurements obtained from the hair matrix. Their results showed higher HCC levels and lower DHEA to cortisol ratios among a high-stress group. The positive association we found between HCC and cortisol/DHEA(S) is consistent with the findings of Qiao et al [23] and lends insight into the neuroendocrine stress variables that may be predictors of HCC. Interestingly, we found a stronger correlation of HCC with cortisol/DHEA(S) rather than cortisol/DHEA; potentially due to the longer half-life and lower clearance of DHEA-S compared with DHEA. Although the variance in HCC attributed to cortisol/DHEA(S) was of small magnitude, the strength of the association may have been greater if the time period and collections (number of days, collections per day) of cortisol and DHEA(S) were extended to more than a single point in time. Previous reports have shown that salivary cortisol has a higher correlation to HCC when the time period of collection is 30 days [30] rather than 1 or 2 sampling days [12].

We also found a weak negative correlation between PSS and the Cortisol/hs-CRP. Chronic stress and depression are associated with reduced integrity of immune responses. Somewhat paradoxically, these mental conditions may be associated with immune suppression or excessive inflammation [31,32]. Cortisol and inflammatory mediators, such as CRP, have a reciprocal relationship such that cortisol is the primary neuroendocrine hormone responsible for keeping inflammation in check, and numerous inflammatory mediators drive the HPA axis leading to an increased level of cortisol. Thus, physical and mental health are somewhat dependent on a balance between cortisol and inflammatory processes, which are necessary for combating infectious challenges. Some researchers have argued that the ratio of cortisol to CRP is a better indicator of physical and mental health than either of these biomarkers alone [32,33].

In terms of clinical applicability, we found a positive association of HCC with obesity, prediabetes and diabetes, cardiovascular disease, and endocrine conditions, and an inverse association with anxiety, which is consistent with previous reports [10,12,34,35]. A recent literature review supports an association between HCC and post-traumatic stress disorder, depressive disorders, and ongoing social and family stress [36]. While the mechanism by which cortisol enters the hair shaft is not fully understood [9], the overlap between the somatic and mental health conditions associated with HCC and the ones associated with plasma cortisol support that the mechanism is primarily via blood.

### Limitations

This study has several additional limitations. The primary limitation in our study was the small sample size, which may have limited the ability of our correlations to reach statistical significance. Second, the cross-sectional design and single point-in-time data collection restrict the implications of our findings. In addition, the predominance of males and recruitment among business executives limit generalizability. The largely male cohort also reduced the feasibility of hair sample collection in the clinical setting. Although we used well-validated surveys of subjective stress and resilience, other self-rated instruments could have been used to assess different stressor categories, such as response to global stress as well as stressful life events. Due to the variability of hair length among participants, different lengths of hair were obtained, representing different durations of hair cortisol accumulation in our weighed samples. Finally, the data collection is not recent.

### Comparison With Previous Work

Although we interpret our results with caution due to the lack of statistical significance, the weak inverse association found between PSS and HCC suggests a potential methodologic advantage of objective stress measures for evaluating stress burden in clinical practice and that retrospective stress perception may not be a sufficient vulnerability marker. This finding is consistent with previous studies that have shown an inconclusive, often contradictory association between perceived stress and HCC [10]. In a review of HCC with subjective stress measures, less than half of the studies reported a significant association [37]. Potential explanations include relatively small sample sizes, heterogeneity of the study populations, diversity of stress questionnaires, and variability in hair lengths. In addition, questionnaire-based assessments may be influenced by awareness about affective states, social desirability, and retrospection bias [38]. Retrospective self-perception of stress may also differ from stress reactivity elicited by contextual factors such as anticipation and social evaluative threat.

Our regression results corroborate the robustness of HCC against potential demographic and hair treatment confounders [10,12]. In a meta-analysis of human studies using HCC, Stalder et al [12] identified a significant positive association between HCC and age, male sex, and less frequent hair washing. Hair treatments were negatively associated with HCC. However, the association with each of these variables was weak [12]. We similarly found a weak association of HCC with age, male sex, hair washing frequency, and use of hair treatments. We were unable to explore the association with race due to the small number of non-White participants in our study sample. In addition to demographic and hair treatment variables, medications may influence plasma cortisol. Similar to our results, they have not been found to be a significant inclusion factor in HCC [10,12].

### Future Directions

Future, larger studies may benefit from the addition of biometric markers in guiding the assessment of stress-related health risk and management strategies. In addition, since cortisol is only one output of the complex HPA axis, the addition of an index of net glucocorticoid may have utility as an adjunct biomarker for chronic stress. More studies are needed to determine the types of psychological stressors and elements of cortisol production and regulation reflected in HCC and potentially identify a complement of neuroendocrine somatic stress biomarkers that are more apt to yield insight into the mechanism of stress-related disease vulnerability than HCC alone.

### Conclusions

While this study was a preliminary exploration into the elements of stress perception and reactivity measured by hair cortisol, further studies are needed to determine the subjective and blood measured correlates measured by hair cortisol and assess its utility in a clinical setting.

## Supplementary material

10.2196/63811 Multimedia Appendix 1 Informed consent.

## Abbreviations:

CD-RISC: Connor Davidson Resilience Scale
DHEA: dehydroepiandrosterone
DHEA(S): collective term for dehydroepiandrosterone and dehydroepiandrosterone-sulfate
DHEA-S: dehydroepiandrosterone-sulfate
ELISA: enzyme-linked immunosorbent assay
HCC: hair cortisol concentration
HPA: hypothalamus-pituitary-adrenal
hs-CRP: high-sensitivity C-reactive protein
PSS: Perceived Stress Scale

## References

[1] Rice DP, Miller LS (1998). Health economics and cost implications of anxiety and other mental disorders in the United States. Br J Psychiatry.

[2] Trautmann S, Rehm J, Wittchen HU (2016). The economic costs of mental disorders: Do our societies react appropriately to the burden of mental disorders?. EMBO Rep.

[3] Kalia M (2002). Assessing the economic impact of stress--the modern day hidden epidemic. Metab Clin Exp.

[4] Murray CJL, Lopez AD (1996). The Global Burden of Disease: A Comprehensive Assessment of Mortality and Disability from Diseases, Injuries, and Risk Factors in 1990 and Projected to 2020 ; Summary.

[5] Cohen S, Janicki-Deverts D, Miller GE (2007). Psychological stress and disease. JAMA.

[6] Sapolsky RM, Romero LM, Munck AU (2000). How do glucocorticoids influence stress responses? Integrating permissive, suppressive, stimulatory, and preparative actions. Endocr Rev.

[7] Schneiderman N, Ironson G, Siegel SD (2005). Stress and health: psychological, behavioral, and biological determinants. Annu Rev Clin Psychol.

[8] Epel ES, Crosswell AD, Mayer SE (2018). More than a feeling: a unified view of stress measurement for population science. Front Neuroendocrinol.

[9] Russell E, Koren G, Rieder M, Van Uum S (2012). Hair cortisol as a biological marker of chronic stress: current status, future directions and unanswered questions. Psychoneuroendocrinology.

[10] Wester VL, van Rossum EFC (2015). Clinical applications of cortisol measurements in hair. Eur J Endocrinol.

[11] Stalder T, Kirschbaum C (2012). Analysis of cortisol in hair--state of the art and future directions. Brain Behav Immun.

[12] Stalder T, Steudte-Schmiedgen S, Alexander N (2017). Stress-related and basic determinants of hair cortisol in humans: a meta-analysis. Psychoneuroendocrinology.

[13] Prado-Gascó V, de la Barrera U, Sancho-Castillo S, de la Rubia-Ortí JE, Montoya-Castilla I (2019). Perceived stress and reference ranges of hair cortisol in healthy adolescents. PLoS One.

[14] Lehrer HM, Steinhardt MA, Dubois SK, Laudenslager ML (2020). Perceived stress, psychological resilience, hair cortisol concentration, and metabolic syndrome severity: a moderated mediation model. Psychoneuroendocrinology.

[15] Charney DS (2004). Psychobiological mechanisms of resilience and vulnerability: implications for successful adaptation to extreme stress. AJP.

[16] Taylor MK (2013). Dehydroepiandrosterone and dehydroepiandrosterone sulfate: anabolic, neuroprotective, and neuroexcitatory properties in military men. Mil Med.

[17] Maninger N, Wolkowitz OM, Reus VI, Epel ES, Mellon SH (2009). Neurobiological and neuropsychiatric effects of dehydroepiandrosterone (DHEA) and DHEA sulfate (DHEAS). Front Neuroendocrinol.

[18] Jin RO, Mason S, Mellon SH (2016). Cortisol/DHEA ratio and hippocampal volume: a pilot study in major depression and healthy controls. Psychoneuroendocrinology.

[19] Morgan CA, Southwick S, Hazlett G (2004). Relationships among plasma dehydroepiandrosterone sulfate and cortisol levels, symptoms of dissociation, and objective performance in humans exposed to acute stress. Arch Gen Psychiatry.

[20] Hechter O, Grossman A, Chatterton RT (1997). Relationship of dehydroepiandrosterone and cortisol in disease. Med Hypotheses.

[21] Cohen S, Janicki-Deverts D, Doyle WJ (2012). Chronic stress, glucocorticoid receptor resistance, inflammation, and disease risk. Proc Natl Acad Sci U S A.

[22] Suarez EC, Sundy JS, Erkanli A (2015). Depressogenic vulnerability and gender-specific patterns of neuro-immune dysregulation: what the ratio of cortisol to C-reactive protein can tell us about loss of normal regulatory control. Brain Behav Immun.

[23] Qiao S, Li X, Zilioli S (2017). Hair measurements of cortisol, DHEA, and DHEA to cortisol ratio as biomarkers of chronic stress among people living with HIV in China: known-group validation. PLoS ONE.

[24] Cohen S, Kamarck T, Mermelstein R (1983). A global measure of perceived stress. J Health Soc Behav.

[25] Cohen S (1988). The Social Psychology of Health, in The Claremont Symposium on Applied Social Psychology.

[26] Cohen S, Janicki‐deverts D (2012). Who’s stressed? Distributions of psychological stress in the United States in probability samples from 1983, 2006, and 2009 ^1^. J Applied Social Pyschol.

[27] Connor KM, Davidson JRT (2003). Development of a new resilience scale: the Connor-Davidson Resilience Scale (CD-RISC). Depress Anxiety.

[28] Sauvé B, Koren G, Walsh G, Tokmakejian S, Van Uum SHM (2007). Measurement of cortisol in human hair as a biomarker of systemic exposure. Clin Invest Med.

[29] (2019). StataCorp, stata statistical software: release 16.

[30] Short SJ, Stalder T, Marceau K (2016). Correspondence between hair cortisol concentrations and 30-day integrated daily salivary and weekly urinary cortisol measures. Psychoneuroendocrinology.

[31] Blume J, Douglas SD, Evans DL (2011). Immune suppression and immune activation in depression. Brain Behav Immun.

[32] Sharpley CF, Bitsika V, McMillan ME, Jesulola E, Agnew LL (2021). Does the cortisol: CRP ratio inform the measurement of individual burden of illness for depression in community samples?. Journal of Affective Disorders Reports.

[33] Bouras M, Roquilly A, Mahé PJ (2019). Cortisol total/CRP ratio for the prediction of hospital-acquired pneumonia and initiation of corticosteroid therapy in traumatic brain-injured patients. Crit Care.

[34] Manenschijn L, Schaap L, van Schoor NM (2013). High long-term cortisol levels, measured in scalp hair, are associated with a history of cardiovascular disease. J Clin Endocrinol Metab.

[35] Feller S, Vigl M, Bergmann MM, Boeing H, Kirschbaum C, Stalder T (2014). Predictors of hair cortisol concentrations in older adults. Psychoneuroendocrinology.

[36] Botschek T, Hußlein V, Peters EMJ, Brosig B (2023). Hair cortisol as outcome parameter for psychological and neuropsychiatric interventions-a literature review. Front Psychiatry.

[37] Staufenbiel SM, Penninx BWJH, Spijker AT, Elzinga BM, van Rossum EFC (2013). Hair cortisol, stress exposure, and mental health in humans: a systematic review. Psychoneuroendocrinology.

[38] Mauss IB, Levenson RW, McCarter L, Wilhelm FH, Gross JJ (2005). The tie that binds? Coherence among emotion experience, behavior, and physiology. Emotion.

