# Supplementary material for: Relationship of Hair Cortisol Concentration With Perceived and Somatic Stress Indices: Cross-Sectional Pilot Study
Source: JMIR Form Res. 2025 Jun 11;9:e63811. doi: 10.2196/63811 (PMC12176311; doi:10.2196/63811)
Supplement: Multimedia Appendix 1 [file formative-v9-e63811-s001.doc]

#

**You Are Being Asked to Be in a Research Study**

## What Is a Research Study?

The main purpose of research studies is to gain knowledge. This knowledge may be used to help others. Research studies are not intended to benefit you directly, though some might.

**Do I Have to Do This?**

**No. Being in this study** **is entirely your choice. If you decide to join this study, you can change your mind later on and withdraw from the research study.**

Taking part in a study is separate from medical care. The decision to join or not join the research study will not affect your status as a patient.

**What Is This Document?**

This form is an informed consent document. It will describe the study risks, procedures, and any costs to you.

This form is also a HIPAA Authorization document. It will describe how your health information will be used and by whom.

Signing this form indicates you are willing to take part in the study and allow your health information to be used.

**What Should I Do Next?**

1. Read this form, or have it read to you.
2. Make sure the study doctor or study staff explains the study to you.
3. Ask questions (e.g., time commitment, unfamiliar words, specific procedures, etc.)
4. If there will be medical treatment, know which parts are research and which are standard care.
5. Take time to consider this, and talk about it with your family and friends.

**Emory University**

**Consent to be a Research Subject / HIPAA Authorization**

**Title**: Physiologic Stress and Epigenetic Age Acceleration

**Principal Investigator:** Sharon Horesh Bergquist, MD, FACP

Assistant Professor of Medicine

Emory School of Medicine

Division of General Medicine & Geriatrics

1365 Clifton Rd, Building A, 1^st^ floor

Atlanta, GA 30322

**Co-Principal Investigator:** Alicia K. Smith, Ph.D.

Associate Professor

Psychiatry and Behavioral Sciences

Emory University School of Medicine

101 Woodruff Circle NE, Ste 4113

Atlanta, GA 30322

## Introduction

You are being asked to be in a medical research study. This form is designed to tell you everything you need to think about before you decide if you want to be a part of the study. **It is entirely your choice. If you decide to take part, you can change your mind later on and withdraw from the research study.** The decision to join or not join the research study will not cause you to lose any medical benefits. If you decide not to take part in this study, your doctor will continue to treat you.

Before making your decision:

- Please carefully read this form or have it read to you
- Please listen to the study doctor or study staff explain the study to you
- Please ask questions about anything that is not clear

You can take a copy of this consent form, to keep. Feel free to take your time thinking about whether you would like to participate. You may wish to discuss your decision with family or friends. Do not sign this consent form unless you have had a chance to ask questions and get answers that make sense to you. By signing this form you will not give up any legal rights.

## What is the purpose of this study?

The purpose of this study is to see how stress interacts with genes to affect the rate of aging. Our environment and lifestyle impact gene expression by turning “on” or “off” certain genes throughout our lifetime. Some of these genes are involved in the aging process. By measuring genetic modifications that control gene expression, called epigenetics, this study will determine each participant’s biologic age. This biologic age can be different than a person’s chronologic age. A novel technique using hair samples will be used to measure chronic stress. This study will one of the first to use these advanced technologies to evaluate the link between chronic stress and biologic age. Medical information and other tests obtained during the executive health physical will also be used to correlate biologic age to early markers of disease as well as disease conditions. We plan to enroll approximately 50 subjects into this study.

## What will I be asked to do?

If you agree to participate in this study, you will be asked to complete two short questionnaires. One is a 10-item questionnaire that will rate how you perceives stress. The other is a 25-item questionnaire that will assess your resilience to stress. Together, these questionnaires will take approximately 5 minutes to complete. You will be asked to have two tubes of blood drawn (2mL each). The blood samples will be processed for 1) genetic changes that help predict your biologic age, or how healthy you are aging and 2) your cortisol and DHEA levels. You will also be asked to have a pencil wide sample of hair taken from the back of your head (approximately 50 hairs), cut as close to the scalp as possible. This hair sample will be used to measure your chronic stress (each centimeter of hair measures a month’s exposure to the stress hormone, cortisol). You can opt out of providing either the blood or hair sample, but you will need to provide one or the other. Providing both is preferred. If you agree to provide a hair sample, you will be asked a few brief questions about use of any hair chemicals or treatments that may influence the study results. The questionnaires, blood, and hair sampling will all take place during the day of your executive physical.

## Who owns my study information and samples?

## If you join this study, you will be donating your samples and study information. You will not receive any compensation if your samples or information are used to make a new product. If you withdraw from the study, data and samples that were already collected may be still be used for this study. Some of the blood samples collected for this study will be placed into smaller samples and frozen. The hair samples will be not retained for any future use.

## What are the possible risks and discomforts?

There may be side effects from the study procedures that are not known at this time.

The most common risks and discomforts expected in this study are:

- Pain, fainting, and possible bruising and infection at the site from which you have your blood drawn
- The very small area in the back of your head from which the hair sample is taken will have shorter hair until it grows back.

Rare but possible risks include:

- Infection at the site from which you have your blood drawn

It is possible that the researchers will learn something new during the study about the risks of being in it. If this happens, they will tell you about it. Then you can decide if you want to continue to be in this study or not. You may be asked to sign a new consent form that includes the new information if you decide to stay in the study.

## Will I benefit directly from the study?

This study is not designed to benefit you directly. This study is designed to learn more about how stress affects how we age, and our risk of developing chronic medical conditions. The study results may be used to help others in the future.

##### Will I be compensated for my time and effort?

You will not be offered compensation for being in this study.

##### What are my other options?

If you decide not to enter this study, it will not affect the medical care that you receive during your executive physical, or during any subsequent visit to The Emory Clinic.

**How will you protect my private information that you collect in this study?**

Whenever possible, a study number, rather than your name, will be used on study records. Your name and other identifying information will not appear when we present or publish the study results.

Study records can be opened by court order. They also may be provided in response to a subpoena or a request for the production of documents.

**Storing and Sharing your Information**

Your samples, genomic data and health information will be stored and shared with other researchers. The samples and information will be available for any research question, such as research to understand what causes certain diseases (for example heart disease, cancer, or psychiatric disorders), development of new scientific methods, or the study of where different groups of people may have come from.

**How is my Genetic Information Protected? What are the Risks?**

The Genetic Information Nondiscrimination Act (GINA) is a federal law that generally makes it illegal for health insurance companies, group health plans, and most employers to discriminate against you based on your genetic information. This law generally will protect you in the following ways:

- Health insurance companies and group health plans may not request your genetic information that we get from this research.
- Health insurance companies and group health plans may not use your genetic information when making decisions regarding your eligibility or premiums.
- Employers with 15 or more employees may not use your genetic information that we get from this research when making a decision to hire, promote, or fire you or when setting the terms of your employment.

Be aware that GINA does **not** protect you against genetic discrimination by companies that sell life insurance, disability insurance, or long-term care insurance, and does not apply to employers with less than 15 employees.

In addition to GINA, the State of Georgia has laws that prohibit insurers from using genetic testing information for any non-treatment purpose. However, like GINA, this state law protection has exclusions: life insurance policies, disability income policies, accidental death or dismemberment policies, medicare supplement policies, long-term care insurance policies, credit insurance policies, specified disease policies, hospital indemnity policies, blanket accident and sickness policies, franchise policies issued on an insurance policy written as a part of workers’ compensation equivalent coverage, or other similar limited accident and sickness policies.

**Privilege**

In the State of Georgia, your genetic information has special legal protections called “privilege,” which means that the information cannot be used as evidence in a court.  By signing this form and allowing us to use and disclose your genetic information for the purposes described in this consent, you waive any privilege with regard to that genetic information, meaning that the information loses this legal protection.

**Medical Record**

If you have been an Emory Healthcare patient before, then you already have an Emory Healthcare medical record. If you have never been an Emory Healthcare patient, you do not have one. An Emory Healthcare medical record will be made for you if an Emory provider or facility gives you any services or procedures for this study.

Copies of the consent form/HIPAA authorization that you sign will be put in any Emory Healthcare medical record you have now or any time during the study.

The results of some study tests and procedures will be used only for research purposes and will *not* be placed in your medical record. For this study, those items include:

- The questionnaire results about your perceived stress and resilience to stress
- Blood test measures of your stress response
- Blood tests that measure how you are genetically aging

Tests and procedures done at non-Emory places may not become part of your Emory medical record. Also, if you decide to be in this study, it is up to you to let your other health providers know.

## Costs

There will be no costs to you for participating in this study, other than basic expenses like transportation. You will not be charged for any of the research activities. If the study procedures (blood draw, hair sample collection) result in any medical complications, the cost of treatment for those complications may be charged to you or your insurance.

## Withdrawal from the Study

You have the right to leave a study at any time without penalty.

The researchers also have the right to stop your participation in this study without your consent for any reason, especially if they believe it is in your best interest or if you were to object to any future changes that may be made in the study plan.

**Authorization to Use and Disclose Protected Health Information**

The privacy of your health information is important to us. We call your health information that identifies you, your “protected health information” or “PHI.” To protect your PHI, we will follow federal and state privacy laws, including the Health Insurance Portability and Accountability Act and regulations (HIPAA). We refer to all of these laws as the “Privacy Rules.” Here we let you know how we will use and disclose your PHI for the main study and for any optional studies in which you may choose to participate.

**Main Study**

**PHI that Will be Used/Disclosed:**

The PHI that we will use or share for the main research study includes:

- Medical information about you including your medical history and present/past medications.
- Results of exams, procedures and tests you have before and during the study.
- Laboratory test results.

**Purposes for Which Your PHI Will be Used/Disclosed:**

We will use and share your PHI for the conduct and oversight of the research study. We will use and share your PHI to conduct normal business operations. We may share your PHI with other people and places that help us conduct or carry out the study, such as laboratories, data management centers, data monitors, contract research organizations, Institutional Review Boards (IRBs) and other study sites. If you leave the study, we may use your PHI to determine your health, vital status or contact information.

**Use and Disclosure of Your Information That is Required by Law**:

We will use and disclose your PHI when we are required to do so by law. This includes laws that require us to report child abuse or abuse of elderly or disabled adults. We will also comply with legal requests or orders that require us to disclose your PHI. These include subpoenas or court orders.

**Authorization to Use PHI is Required to Participate**:

By signing this form, you give us permission to use and share your PHI as described in this document. You do not have to sign this form to authorize the use and disclosure of your PHI. If you do not sign this form, then you may not participate in the research study. You may still receive non-research related treatment.

**People Who Will Use/Disclose Your PHI:**

The following people and groups will use and disclose your PHI in connection with the research study:

- The Principal Investigator and the research staff will use and disclose your PHI to conduct the study.
- Emory may use and disclose your PHI to run normal business operations.
- The Principal Investigator and research staff will share your PHI with other people and groups to help conduct the study or to provide oversight for the study.
- The following people and groups will use your PHI to make sure the research is done correctly and safely:
  - Emory offices that are part of the Human Research Participant Protection Program and those that are involved in study administration and billing. These include the Emory IRB, the Emory Research and Healthcare Compliance Offices, and the Emory Office for Clinical Research.
  - Government agencies that regulate the research.
  - Public health agencies.
  - Research monitors and reviewer.
  - Accreditation agencies.
- Sometimes a Principal Investigator or other researcher moves to a different institution. If this happens, your PHI may be shared with that new institution and their oversight offices. PHI will be shared securely and under a legal agreement to ensure it continues to be used under the terms of this consent and HIPAA authorization.

**Optional Storage of Data/Specimens for Future Research:**

**PHI That Will be Used/Disclosed for Optional Study:**

The PHI that we will use and/or disclose (share) for the optional storage and future use of your PHI includes: blood samples and blood tests, medical information about you, including medical history and other test results.

**Purposes for which your PHI will be Used/Disclosed for Optional Study:**

We will use and disclose your PHI for the conduct and oversight of the optional storage and future research use of your PHI.

**Authorization for This Use of PHI is Required to Participate in Optional Study, but Not in Main Study**:

You do not have to authorize the use and disclosure of your PHI. If you do not authorize the use and disclosure of your PHI for the optional study, then you may not participate in the optional research study. You can still be in the main research study even if you don’t participate in the optional study.

**People Who Will Use/Disclose Your PHI for Optional Study:**

The following people and groups will use and disclose your PHI in connection with the optional storage and future research use of your PHI:

- The same people and groups who will use and disclose your PHI for the Main Study will also do so in connection with the optional research study/storage of PHI for future research.
- In addition, future researchers may also use and disclose your PHI for the Optional Study.

**Expiration of Your Authorization**

Your PHI will be used until this research study ends.

**Revoking Your Authorization**

If you sign this form, at any time later you may revoke (take back) your permission to use your information. If you want to do this, you must contact the study team at:

Emory Executive Health

1365 Clifton Rd.

Building A, 1^st^ floor

Atlanta, GA 30322

(404) 778-1234

At that point, the researchers would not collect any more of your PHI. But they may use or disclose the information you already gave them so they can follow the law, protect your safety, or make sure that the study was done properly and the data is correct. If you revoke your authorization you will not be able to stay in the main study.

**Other Items You Should Know about Your Privacy**

Not all people and entities are covered by the Privacy Rules. HIPAA only applies to health care providers, health care payers, and health care clearinghouses. If we disclose your information to people who are not covered by the Privacy Rules, including HIPAA, then your information won’t be protected by the Privacy Rules. People who do not have to follow the Privacy rules can use or disclose your information with others without your permission if they are allowed to do so by the laws that cover them. For this study, there are no people or companies involved that are not covered by the Privacy Rules.

To maintain the integrity of this research study, you generally will not have access to your PHI related to this research until the study is complete. When the study ends, and at your request, you generally will have access to your PHI that we maintain in a designated record set. A designated record set is data that includes medical information or billing records that your health care providers use to make decisions about you. If it is necessary for your health care, your health information will be provided to your doctor.

We may remove identifying information from your PHI. Once we do this, the remaining information will not be subject to the Privacy Rules. Information without identifiers may be used or disclosed with other people or organizations for purposes besides this study.

**Contact Information**

Contact Sharon Bergquist, MD at 404-778-1234:

- if you have any questions about this study or your part in it,
- if you have questions, concerns or complaints about the research

Contact the Emory Institutional Review Board at 404-712-0720 or 877-503-9797 or [irb@emory.edu](mailto:irb@emory.edu):

- if you have questions about your rights as a research participant.
- if you have questions, concerns or complaints about the research.
- You may also let the IRB know about your experience as a research participant through our Research Participant Survey at <http://www.surveymonkey.com/s/6ZDMW75>.

## Consent and Authorization

**Consent and HIPAA Authorization for Optional Study/Studies:**

Please initial below if you opt to participate in and authorize use and disclosure of your PHI for the future storage and research use of your PHI:

**[OPTIONAL STORAGE OF DATA/SPECIMENS FOR FUTURE USE] ____________Initials**

***TO BE FILLED OUT BY SUBJECT ONLY***

Please **print** your name, **sign**, and **date** below if you agree to be in the main study. By signing this consent and authorization form, you will not give up any of your legal rights. We will give you a copy of the signed form to keep.

**Name of Subject**

**Signature of Subject (18 or older and able to consent) Date Time**

***TO BE FILLED OUT BY STUDY TEAM ONLY***

**__________________________________________________________**

**Name of Person Conducting Informed Consent Discussion**

**Signature of Person Conducting Informed Consent Discussion Date Time**
